# Editorial: The role of metabolomics in ART: from diagnosis to treatment

**DOI:** 10.3389/fendo.2025.1558561

**Published:** 2025-02-05

**Authors:** Mara Simopoulou, Sokratis Grigoriadis, Evangelos Maziotis, Dragoş Creţoiu, George Mastorakos, Roger Sturmey

**Affiliations:** ^1^ Department of Physiology, Medical School, National and Kapodistrian University of Athens, Athens, Greece; ^2^ Carol Davila University of Medicine and Pharmacy, Bucharest, Romania; ^3^ Unit of Endocrinology, Diabetes mellitus, and Metabolism, Aretaieion Hospital, Medical School, National and Kapodistrian University of Athens, Athens, Greece; ^4^ Biomedical Institute for Multimorbidity, Hull York Medical School, University of Hull, Hull, United Kingdom

**Keywords:** assisted reproduction (ART), *in vitro* fertilization - embryos, metabolomics, biomarkers, reproductive physiological processes

In the 46 years since Louise Brown’s birth, rapid advances in medically assisted reproduction (MAR) have led to the birth of more than 12 million children to date. This has meant that, millions of couples have been able to overcome infertility achieving their desired family planning. These numbers, while impressive, seem small compared to future projections. Current estimates suggest that 1 in 6 couples of reproductive age are currently infertile, a prevalence that is expected to increase in the coming years. Extrapolating infertility rates and uptake of MAR, it is predicted that by 2100, 76 years from now, the number of children born through *in vitro* fertilization (IVF) will reached more than 167 million births (1–3).

Given that a significant percentage of the world’s population will be born through IVF in the coming years, new challenges and needs arise for optimizing the health services provided through IVF. Since the advent of IVF, the scientific community has aimed to *optimize* methods, conducting research that uses outcome measure parameters such as implantation rate, clinical pregnancy rate (CPR) and live birth rate (LBR). The results of these efforts have been impressive, with LBR per embryo transfer now reaching 30% (4). Although efforts to maximize LBR are important, scientific interest is beginning to shift to other equally important outcome measures, such as minimizing the time from diagnosis of infertility to achieving the desired reproductive outcome and ensuring the long-term health of offspring born via IVF (5). This trail of scientific thought needs sophisticated and novel approaches enabling the holistic investigation of reproductive physiology and pathophysiology. In the era of ‘Omics and Big Data, the introduction of novel techniques into reproductive medicine seems to be essential in order to identify novel diagnostic and therapeutic management strategies (6).

A case in point is the study by Gao et al. in which lipidomic analysis was performed on follicular fluid obtained from cases with an expected hyper-response to ovarian stimulation. Using state-of-the-art lipidomic analysis by electrospray ionization high-resolution mass spectrometry, the research team discovered that cases that eventually develop ovarian hyperstimulation syndrome (OHSS) had a characteristic lipidomic profile. Thislipidomic profile was characterized by increased levels of esters and decreased levels of lysophosphatidylcholine, phosphatidylinositol, sphingomyelin, dimethylphosphatidylethanolamine and lysodimethylphosphatidylethanolamine. These data are significant and may unlock new understanding of the complex pathophysiology of the OHSS phenomenon. In addition, studies such as this one highlight the importance of personalizing patient management during ovarian stimulation and ultimately contribute to our efforts to develop safe ovarian stimulation strategies in expected hyper-responder cases, with the ultimate target of minimizing the incidence of OHSS phenomenon, which remains probably the most serious complication associated with IVF (7).

A different perspective was offered by study by Ma et al., which highlights the significant contribution of metabolomics in our efforts to provide robust evidence regarding the efficacy of numerous interventions aimed at improving the reproductive dynamics of infertile patients. The authors conducted a double-blind, randomized, placebo-controlled trial recruiting 119 infertile patients undergoing IVF to investigate the efficacy of Guilu Erxian Ointment in improving reproductive outcome in patients with poor ovarian response due to advanced maternal age. In addition to analyzing key reproductive outcomes, the authors performed ultra-high performance liquid chromatography-triple quadrupole mass spectrometry (UHPLC-QTRAP MS) analysis on follicular fluid samples obtained from poor responders treated with Guilu Erxian ointment, poor responders treated with placebo, and healthy young controls. Targeted UHPLC-QTRAP MS analysis revealed significant similarities in the follicular fluid metabolome between the Guilu Erxian Ointment treated group and the normal young group. Interestingly, the metabolomic profiles of the aforementioned groups differed from the corresponding metabolomic profile observed in the placebo group. Furthermore, using this high throughput metabolomic analysis, the authors were also able to identify the potential mechanisms by which Guilu Erxian Ointment may improve ovarian dynamics in cases of poor ovarian response, suggesting the beneficial effect of Guilu Erxian Ointment on amino acid and lipid metabolism.

The study of Ma et al. highlights the importance of metabolomic research in our efforts to improve ovarian functionality in cases of poor ovarian response and diminished ovarian reserve. Questions remain unanswered on the efficacy and safety of many proposed approaches aimed at the rejuvenation of ovarian function, with platelet rich plasma intraovarian infusion and stem cell therapy being typical examples (8–10). This study perfectly showcases the value of integrating metabolomics into research, as it offers the possibility to test in each case both the efficacy and the potential physiological mechanisms through which each intervention affects ovarian functionality (11, 12).

In addition to the beneficial impact exerted by introducing sophisticated metabolomic profiling into modern reproductive medicine research, targeted research on hormones that affect the metabolism of reproductive organs is also required. The driver behind that is the need for understanding how these hormones are associated with the quality of gametes, embryos and other organs and tissues such as the endometrium, all of which are critical for achieving and successfully maintaining a pregnancy. Although this research field has been identified as a focal point for many decades, unanswered questions that are not typically considered in the day-to-day clinical practice, still remain.

A typical paradigm is the desirable estradiol levels that should be achieved for initiating GnRH antagonist administration in conventional ovarian stimulation protocols. In the retrospective study by Wang et al., in which the results of 1.493 IVF cycles were analyzed, the value of continuous monitoring of estradiol levels during ovarian stimulation was highlighted in the clinical decision making for the optimal timing of GnRH antagonist administration. The researchers demonstrated that administration of a GnRH antagonist leads to optimal clinical outcomes when estradiol levels are between 436.8-658.6 pg/ml. Importantly, the researchers observed extremely adverse outcomes of IVF cycles when GnRH antagonist administration is performed in cases where estradiol levels are > 894.4 pg/ml. These observations are of added benefit for clinical practice. If these data are independently verified it may indicate IVF cycle cancelation, not only when endometrial thickness fails to meet the optimal target of > 7 mm, but also when estradiol levels are too high, corresponding to abnormal ovarian hyper activation (13).

Along the same lines, the study by Li et al. highlighted the need for progesterone monitoring for clinical decision making regarding the timing of ovulation triggering in IVF frozen embryo transfer (FET) cycles. The authors performed a retrospective data analysis of more than 500 cases undergoing preimplantation genetic testing FET cycles. The results suggest that progesterone levels > 1.5 ng/ml on the trigger day are strongly associated with adverse outcomes, such as reduced clinical pregnancy and live birth rates, in subsequent FET cycles. These findings merit further investigation to better understand the effects of high progesterone levels on oocyte and embryo metabolism and developmental potential, since progesterone levels on the day of ovulation triggering are not commonly evaluated in daily clinical practice (13, 14).

Beyond the impact of progesterone levels on the day of ovulation triggering on oocyte and embryo quality, there is an incentive to further elaborate an optimal protocol design for luteal phase support. Several protocols for luteal phase support have been introduced regarding IVF fresh or frozen embryo transfer cycles (15, 16). However, questions remained unanswered with regards to the optimal strategy in intrauterine infusion (IUI) MAR cases. A large systematic review and meta-analysis published by Casarramona et al. indicate that proper luteal phase support is also required for IUI. More specifically, results provided by twelve randomized controlled trials (RCTs) involving more than 2,600 patients indicate a significant increase of clinical pregnancy and live birth rates in IUI cases receiving luteal phase support, especially when ovarian stimulation is achieved via gonadotropin administration. These findings are of significance since proper luteal phase support in IUI cycles may be associated with improved reproductive outcomes. This may enable avoidance of IVF overuse, a far more invasive management strategy compared to IUI (17).

Considering optimal management strategies for efficiently addressing infertility it is of paramount importance to delve into other pathophysiological conditions directly affecting reproductive system functionality and thus patient performance in the context of IVF. It is well established that chronic stress and low-grade inflammation directly affect homeostasis and metabolism and thus the functionality of several systems and organs, including the reproductive system. Considering the effect of low-grade inflammation on reproductive system functionality, the study by Zhang et al. provides interesting observations supporting that high human serum CRP levels (>3 mg/L) are associated with adverse IVF outcomes. This retrospective data analysis involving 875 women undergoing IVF, demonstrated that in cases of high human serum CRP levels (>3 mg/L) live birth rate is significantly reduced from 53.8% to 39.8%. These findings may suggest the importance of adding CRP level evaluation in the basic infertility investigation, since it has been performed for other hormones, including TSH. Identification of infertile patients suffering from underlying chronic stress and low-grade inflammation prior to MAR may also lead to the optimization of MAR outcomes (18).

In conclusion, optimal management of infertile patients requires an in-depth study and comprehensive analysis of the pathophysiological conditions leading to infertility, elaborating also into the impact of *in vitro* culture conditions on the health of future offspring, in the era of modern personalized and evidence-based clinical practice. The application of metabolomics allows the systemic study of metabolism and homeostasis, which allows clinical practice to be tailored to the specific needs of each case. Therefore, Omics’ technology, and more specifically metabolomics, is a prime example of how translational research can lead to medical personalization of IVF, challenging traditional claims supported by empirical clinical practice.
